# Systemic anticancer therapy (SACT) for lung cancer and its potential for interactions with other medicines

**DOI:** 10.3332/ecancer.2017.764

**Published:** 2017-09-04

**Authors:** Ryan Panchal

**Affiliations:** Imperial College London, Exhibition Road, London SW7 2AZ, UK

**Keywords:** systemic anticancer therapy, clinical audit, lung cancer, medicines reconciliation, drug–drug interactions

## Abstract

**Background:**

Systemic anticancer therapy, comprising chemotherapy agents alongside targeted therapies and immunotherapy, is clinically indicated for late-stage lung cancer. It is delivered in regimens often containing multiple anticancer agents as well as supportive care medicines to reduce side effects, raising potential for polypharmacy and therefore the possibility of drug–drug interactions with medicines taken for comorbidities. A pharmacy-led process commonly performed to assist safe prescribing in secondary care is medicines reconciliation; its benefit in minimising interactions involving systemic anticancer therapy medicines has not been assessed previously.

**Objectives:**

The objectives were to characterise the potential drug–drug interactions between systemic anticancer therapy medicines for lung cancer and other medicines and to evaluate the rate of medicines reconciliation being performed and the extent of documentation of potential interactions (clinical audit).

**Methodology:**

This retrospective case series study involved recording the medicines being taken by lung cancer patients undergoing systemic anticancer therapy elicited in consultations at Chelsea and Westminster Hospital, United Kingdom. Potential interactions were identified and characterised in terms of severity using the British National Formulary and other sources. Patient consultation records were also searched for documentation of medicines reconciliation and acknowledgement of potential drug–drug interactions.

**Results:**

Twenty-three patients were included in this study. Eighty-eight potential drug–drug interactions were identified across 21 patients, 39% (34/88) of which involved the supportive care medicine dexamethasone. 3.0% of consultations included a documented medicines reconciliation, and 15.9% of potential interactions were documented in the notes, with no correlation between the two. Potentially serious interactions were significantly more likely to be documented (*p* < 0.05).

**Conclusions:**

Many potential drug–drug interactions involving anticancer agents and supportive care medicines exist; particular attention should be paid to dexamethasone. Documentation of interactions and medicines reconciliation occur much less often than expected, suggesting there is scope for implementing methods of safe prescribing to prevent adverse drug effects.

## Introduction

### Systemic anticancer therapy (SACT)

There are many different treatment options for lung cancer, and these are influenced by type, extent and progression of disease. These treatments can be categorised into surgery, radiotherapy and systemic anticancer therapy (SACT), combinations of which are also possible. While surgery and radiotherapy tend to be of use in local, early-stage disease, SACT is largely used as first-line treatment in advanced stage IIIb-IV non-small-cell lung cancer (NSCLC), with extensive nodal involvement and metastases, to ‘improve survival, disease control and quality of life’ and is the preferred treatment for both limited and extensive small-cell lung cancer (SCLC) [1]. It is planned in cycles, between which the patient is reviewed for response to therapy.

SACT can be divided into three categories, based on mechanism of action in treating cancer. The most extensive group, chemotherapy, involves the use of cytotoxic drugs to directly destroy tumour cells. It has multiple applications in NSCLC, due to the various intentions of use across most stages of lung cancer. As an adjunct or in combination with radiotherapy, it can be considered pre-operatively in early-disease (stage I–II) patients suitable for surgery; adjuvant chemotherapy aiming to kill cancer cells following radiotherapy is an option in stage II–III disease [2]. There are multiple classes of chemotherapy drug available, with a variety of intracellular targets such as DNA and microtubules. Biological therapies, the second group, are anticancer agents that prevent the spread of cancer by ‘interfering with specific molecules involved in tumour growth and progression’ [3], as opposed to killing tumour cells directly. These targeted therapies have further developed as a result of advancements in tumour analysis for protein mutations that cause uncontrolled proliferation, such as those in epidermal growth factor receptor (EGFR) and anaplastic lymphoma kinase (ALK) [4]. The third mechanism of SACT is immunotherapy, where monoclonal antibodies recruit the immune system to recognise and attack malignant cells [5]. Table 1 details the SACT agents recommended for use in lung cancer.

**Figure 1.**
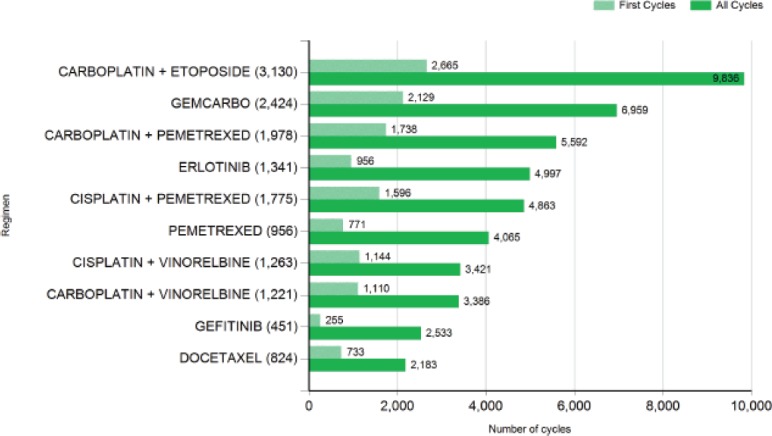
Most common SACT regimens for lung cancer between January and December 2014. Key: GEMCARBO=gemcitabine + carboplatin. Taken from [16].

Application of SACT involves the use of combination therapies. The most recent statistics of the commonest regimens for lung cancer are shown in Figure 1.

Supportive care medicines are often prescribed as part of the SACT regimen to prevent side effects from occurring. These include various antiemetics such as aprepitant, domperidone, and ondansetron, to combat nausea and vomiting, a common adverse effect of most anticancer agents [6]. Notably, some have higher potential for nausea than others, particularly cisplatin, and different regimens thus require varying doses of antiemetic [17]. Corticosteroids, such as dexamethasone, also counter nausea but additionally reduce the incidence and severity of skin rash, another frequent adverse reaction [6]. Other medicines include folic acid and vitamin B12 for the antifolate drug pemetrexed [18, 19] and hydration guidelines for the nephrotoxic platinum-based compounds [20], encompassing combinations of oral furosemide, intravenous saline, and magnesium or potassium salt solutions [21, 22].

## Drug–drug interactions

As discussed, cancer patients for whom SACT is indicated take a multitude of medicines, either within a complex regimen or due to a range of supportive therapies [23]. This increases the likelihood of drug–drug interactions (DDIs), where concurrent administration of two drugs allows one to influence the activity of another [24]. Moreover, interlinking these factors, older patients are the typical demographic of cancer and thus are more likely to be taking multiple regular medicines due to increased comorbidities [25–27].

DDIs, including those involving SACT medicines, can be beneficial and lead to a synergistic (augmented) effect of both drugs, a concept utilised in combination anticancer therapy [28]. However, there is potential for negative outcomes; due to the large number and difference in mechanisms of action between anticancer agents, there are a multitude of adverse DDIs involving chemotherapy drugs [28, 29]. Importantly, several SACT agents, including cisplatin, crizotinib, and EGFR tyrosine kinase inhibitors (TKIs), are CYP450 substrates, meaning they can influence hepatic metabolism of many drugs [29, 30]. Other possible DDIs include platinum-based compounds with nephrotoxic agents such as NSAIDs, a synergistic interaction causing impaired renal function [31], and EGFR-TKIs with antacids, which raise stomach pH and thus prevent absorption of the anticancer agent [32].

The impact of DDIs directly on healthcare is poorly characterised. However, the broader group of adverse drug reactions (ADRs) place a significant strain on patients and hospitals, accounting for 6.5% of all hospital admissions, with a total cost per year of over £500,000 to the NHS [33, 34]. The proportion of this attributed to DDIs is debatable; some suggest an increased risk of readmission related to DDIs [35], particularly in the elderly [36], while other reviews propose the opposite [37, 38]. Nevertheless, as the potential for DDIs in SACT patients is high, methods to increase awareness are crucial to minimise risk of adverse events with which they are associated [39, 40].

### Medicines reconciliation

With regard to safe medicine prescribing, it is important that healthcare professionals (HCPs) are conscientious in maintaining the efficacy of medicines, in order for patients to get the best out of their treatment [27, 41]. This concept is known as medicines optimisation and comprises four guiding principles to improve patient outcome, as outlined by the Royal Pharmaceutical Society [42]: aiming to understand the patient’s experience; evidence-based choice of medicines; ensuring medicines use is as safe as possible; and making medicines optimisation part of routine practice. A major aspect of medicines optimisation that contributes to these aims is medicines reconciliation (Med-Rec), the process of ensuring the medicines a patient is taking are correctly documented [43].

This involves ‘creating and maintaining the most accurate list possible’ of the patient’s medicines, and then ‘comparing… with the current list in use, recognising any discrepancies, and documenting any changes, thereby resulting in a complete list of medicines, accurately communicated’ [27, 44], as defined by the Institute of Healthcare Improvement (IHI). These tasks can be summarised into three elements to form ‘reliable’ reconciliation: verification of the list of current medicines; validation (a review of the current medicines by a trained and competent HCP, noting whether to continue or alter any doses); and clarification, where the current list is compared with the prescribed ‘medication order’ [41, 44].

Previous studies highlight the potential for problems in healthcare without a formal Med-Rec procedure; unintentional discrepancies were found in 70% of medicines prescribed on admission (covering 60% of patients) in a large systematic review by Garfield [41, 45]. Actively implementing the process is also found to be beneficial; it decreases the rate of ‘medication errors’ by 70% and ADRs by 15% [44, 46, 47] in one hospital setting, while another trial found that it reduced potential ADRs by 80% [48]. This suggests Med-Rec is an important part of preventing harm to patients.

### Rationale for study

As clarified, there is a considerable risk of DDIs occurring between anticancer agents, plus various supportive care medicines as part of SACT regimens, and other medicines being taken by patients. The complexity of regimens in lung cancer specifically, utilising drugs with various mechanisms, adds to the potential risk of harm. It was therefore of interest to characterise the severity of these potential DDIs (PDDIs), and review whether established processes of Med-Rec, or simply documentation of PDDIs, could have a role in preventing harm. Numerous studies report aspects of these separately: DDIs involving general chemotherapy have been identified retrospectively in several studies [49–55]; while outcomes of pharmacy-led intervention with Med-Rec [39, 56–62] have also been analysed. However, combining severity of PDDIs and improvement of patient safety has not been carried out previously.

## Aims, objectives and standards

### Aims

The aim is to evaluate the potential for DDIs between medicines in SACT regimens and other medicines taken by lung cancer patients treated at Chelsea and Westminster Hospital (CWH).

### Objectives

1. To identify and characterise PDDIs present between SACT medicines (comprising anticancer agents and supportive care medicines) and other medicines taken by the patients.
2. To assess the process of Med-Rec and evaluate documentation of PDDIs by prescribing HCPs (clinical audit).

### Standards for clinical audit

1. 100% of consultations with a prescribing HCP since the patient started their current SACT regimen include a documented Med-Rec.
2. 100% of PDDIs between SACT medicines and other medicines had been acknowledged and documented by a prescribing HCP.

## Methodology

### Study design, inclusion, and exclusion criteria

This was a single-centre, retrospective case series study. Patients were selected for inclusion if diagnosed with lung cancer and undergoing a SACT regimen as of 31st March 2016 under the care of the Oncology team (led by Professor Mark Bower and Dr Tom Newsom-Davis) at CWH.

### Data collection

For each patient, every instance of documented patient contact at CWH during their SACT regimen was compiled from the patient records. The chosen start point was the last consultation with a prescribing HCP before starting the current SACT regimen in which a full medicines history was taken. For patients on a maintenance SACT regimen, data were recorded from the last consultation in which a full medicines history was elicited before starting the SACT regimen. The endpoint was the cut-off date of 31st March 2016. Data recorded for each consultation include documentation of medicines being taken and changes to the medicine profile as a result of the consultation.

### Identifying PDDIs

A ‘drug chart’ overviewing how the medicine profile changed for each patient during their SACT regimen was then created from the data collection spreadsheet (Appendix 1). PDDIs were identified using three primary sources: the British National Formulary (BNF); Summary of Product Characteristics (SPC) at www.medicines.org.uk; and the London Cancer Alliance (LCA) protocols for each SACT regimen. Only PDDIs involving a SACT medicine—either the anticancer agent, or any prescribed supportive care medicine—were noted, and not between any two non-SACT medicines taken concurrently. Component drugs within a preparation were studied individually.

### Assessment of Med-Rec and documentation of PDDIs

The same patient consultation records were analysed for documentation both of Med-Rec being performed and of any DDIs by prescribing HCPs.

### Definitions

‘PDDI’ (potential drug–drug interaction) is defined as a possible DDI between two medicines (as reported in the BNF, SPC, or LCA protocols) that may have occurred when the patient was taking both concurrently.

‘Anticipated DDI’ is defined as a possible DDI that was identified but did not occur due to intervention before both medicines were being taken simultaneously. There is therefore no possibility of an adverse event due to this DDI occurring.

A DDI is ‘identified’ if noted by the author during retrospective analysis of the collected data; ‘acknowledged’ or ‘documented’ DDIs are those identified and written down during a consultation by a prescribing HCP.

## Results

Twenty-three patients met the criteria for inclusion in this study. SACT regimens being followed, listed by route of administration, are shown in Table 2.

### Identified PDDIs

**Figure 2.**
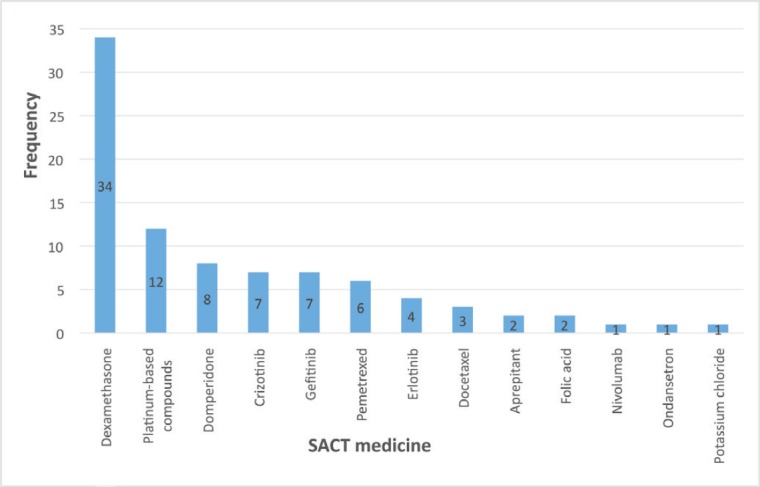
Frequency of PDDIs (total n = 88) for each of the 13 SACT medicines that had at least one identified PDDI.

A total of 88 instances of PDDIs involving SACT medicines across 21 patients were identified. This total includes anticipated DDIs (n = 13). Figure 2 presents the SACT medicines with at least one identified PDDI.

In order to present these data qualitatively, PDDIs were grouped based on effect of interaction and mechanism of the interacting medicine. These 30 distinct DDIs are summarised in Table 3.

The most common identified DDI was that of dexamethasone + antihypertensives (12.5%), followed by: platinum-based compounds + nephrotoxic drugs (8.0%); crizotinib + UGT substrates, dexamethasone + antidiabetics; pemetrexed + nephrotoxic drugs (each 6.8%). The remaining PDDIs had five or fewer instances. In terms of severity, there were six ‘potentially serious’ DDIs, equating to 8.0% of the total number.

### Medicines reconciliation and documentation of PDDIs

Standard 1: 100% of consultations with a prescribing HCP since the patient started their current SACT regimen include a documented Med-Rec.

Outcome 1 (standard not met): 3.0% of consultations with a prescribing HCP since the patient started their current SACT regimen include a documented Med-Rec.

A total of 480 instances of documented patient contact were recorded across all 23 patients. Forty-eight of these were excluded from further analysis: eight were phone calls, three were radiology scan appointments, and 37 were consultations with non-prescribing HCPs.

Of the remaining 432 instances, 13 (3.0%, across 7 patients) included a documented Med-Rec. Additionally, 95.7% of patients had at least one full medicines history elicited by a prescribing HCP. This translates to a full medicines history being taken in 11.8% of consultations.

Standard 2: 100% of identified PDDIs between SACT medicines and other medicines had been acknowledged and documented by a prescribing HCP.

Outcome 2 (standard not met): 15.9% of identified PDDIs between SACT medicines and other medicines had been acknowledged and documented by a prescribing HCP.

Of the 88 instances of PDDIs, 14 (15.9%, across 8 patients) were acknowledged and documented. These are summarised in Table 4.

Comparison of severity and probability of documentation was performed using Fisher’s exact test. Instances of PDDI with an unknown severity (*n* = 30 across both documented and non-documented DDIs) were omitted. Using a standard alpha-level of 0.05, the null hypothesis can be rejected (d.f.=1, two-tailed *p* = 0.019), indicating that potentially serious DDIs were significantly more likely to be documented.

Documented PDDIs were also analysed in relation to Med-Rec processes. None of the 14 PDDIs were acknowledged by a prescribing HCP in the same consultation as a documented Med-Rec. Two (14.3%) were elicited after a full medicines history was taken in the same consultation.

Thirteen of the 14 documented PDDIs can be considered anticipated DDIs, where intervention took place before the interaction could occur. The remaining one instance, gefitinib + warfarin, was acknowledged and documented after both medicines were taken concurrently.

## Discussion

### Identified PDDIs

The wide-ranging nature of this study means there are several different aspects suitable for analysis. Firstly, the variety of SACT regimens—10 in total—confirm the multitude of treatment options for late-stage lung cancer patients and compare reasonably well with national statistics [16].

Regarding the interactions, nearly all patients (91.3%) had at least one identified PDDI, highlighting the importance of characterising them well. Of particular significance were the PDDIs involving supportive care medicines, which accounted for 55% of the total. Most previous studies looking at DDIs involving chemotherapy only consider the anticancer agent [50, 51, 54], although van Leeuwen did report supportive care medicines to be involved in 86% of all identified PDDIs for the cohort studied [55], further supporting their clinical relevance in DDIs.

The major SACT medicine of note here was dexamethasone, which comprised 39% of the total number. Moreover, two further PDDIs were identified involving dexamethasone prescribed separately from the SACT regimen. DDIs with corticosteroids have been reported in the literature—van Leeuwen’s retrospective study found that dexamethasone was the major supportive care medicine involved [55], and Lam considered it a ‘cancer-related drug’ with a variety of mechanisms of interaction [49]. Additionally, a quarter of PDDIs involving dexamethasone were classified as potentially serious. This raises the question of its safety, particularly as it was a prescribed component of all intravenous combination SACT regimens for these patients (Appendix 2). However, regimens are carefully designed in terms of dose, timing, and frequency to minimise harm, with low-dose dexamethasone being taken for only three days for every cycle in these LCA-approved regimens. Nevertheless, caution should be exercised considering the wide range of drugs with which it potentially interacts, especially antihypertensives, which was the single-most common PDDI. Indeed, the high frequency reflects the high prevalence of hypertension in the population, particularly the elderly [63] and therefore suggests it is an important comorbidity to consider.

The next most commonly involved SACT medicine were the platinum-based compounds. This meant that cisplatin and carboplatin were the anticancer agents with the most PDDIs, a finding corroborated by Mouzon in a retrospective study [50] and a review by Scripture [29]. All PDDIs for these were linked with nephrotoxicity, which was also the potential outcome in the interactions involving pemetrexed, another nephrotoxic anticancer agent. Indeed, combining the PDDIs for all three makes kidney damage the most common potential outcome (20.4%). This is particularly dangerous because patients tend to be older and are more likely to have existing renal impairment [64], putting them at greater risk.

Other notable interactions are the remaining potentially serious PDDIs. Although only six of the 30 were classified in this way, each with low frequencies and thus constituting only 8.0% of the total PDDIs, possible outcomes such as increased risk of bleeding and adrenal suppression mean they should be actively avoided. It is also important to note that 10 PDDIs (totalling 27 instances) did not have a classified severity because they were identified from sources other than the BNF, and so any number could also be potentially serious DDIs, limiting the validity of the results.

### Medicines reconciliation and documentation of PDDIs

The extent of identified interactions, with each patient on average having four PDDIs involving SACT medicines, highlights the necessity of safe prescribing and maintaining patient safety. Clinical audit was an effective method to measure this, identifying results as below expected. For the first standard, a remarkably low value of 3.0% of consultations including a documented Med-Rec implies there is almost a complete lack of the process occurring. Indeed, this was the case: the 13 documented Med-Recs were only performed when a patient was discharged from CWH, and never in clinic. UK and US guidelines focus primarily on Med-Rec for inpatients, with guidance stating it should be performed for any ‘transfer of care’, either on admission to hospital, during transfer between wards or on discharge [27]. There is no formally established procedure for patients in ambulatory care at CWH, including this cohort of lung cancer patients attending regular Oncology clinics.

This implies there is scope for implementation of Med-Rec processes for outpatients. IHI suggest collecting a full medicines history and then reviewing if there have been any changes during the consultation, after which a list can be kept ‘on file’ and verified for each subsequent appointment [65]. Several intervention studies have assessed the advantages of reminders for patients to bring in their regular medicines to clinic followed by correction of the medicines list by patients themselves [66–68], all with encouraging results. A further justification to perform Med-Rec for these patients is that attending clinic could be seen as a ‘temporary’ transfer of care, during which the processes of Med-Rec should be carried out.

These shortcomings are also reflected in the second standard regarding PDDI documentation. This again fell well below 100%, suggesting prescribing HCPs do not regularly review the safety of medicines being taken by the patient. Moreover, some were acknowledged simultaneously, meaning the 14 documented PDDIs were across eight consultations only. This below-standard practice could be due to the assumption made by HCPs that reviewing medicines comes under the remit of Pharmacy; involving pharmacists in a multidisciplinary team to ensure accurate medicines lists could improve the documentation of PDDIs and thus prevention of interactions, as seen in Lopez-Martin’s single-centre study in Oncology outpatients [61].

While increasing documentation of PDDIs is clearly key, there were some interesting patterns in the acknowledgement of certain categories. The results revealed the potentially serious interactions were significantly more likely to be documented than non-serious PDDIs. There are also three classes of interacting medicine that constitute the majority (71.4%) of the documented interactions: anticoagulants, PPIs, and antiretrovirals. This suggests that there may be awareness about certain types of DDI involving specific drugs, without the need for research.

Finally, combining the aspects of the study, it appears that performing Med-Rec does not determine whether PDDIs are documented, which was reasonably assessed by the occurrence of both processes in the same consultation. However, this does not take into account the fact that Med-Rec may not always be carried out in a consultation, even though face-to-face Med-Rec is recommended in local guidelines [41]; it could feasibly be done by comparing to the current medicines list without patient input. This means that there is possible underreporting of Med-Rec, potentially skewing results. Despite this, even just ‘verification’ correlates poorly (14.3%) with documentation of PDDIs, suggesting that the majority of acknowledged PDDIs were independent of the taking of a full medicines history in that consultation. Thus, it is beneficial to look at some of the many techniques already established to improve identification of potential interactions, such as the pharmacist-led PINCER [69], the screening tools for elderly patients STOPP/START [70], and My Medicines Passport, a patient-oriented booklet for recording medicines launched in CWH [71].

## Conclusion

In summary, this study provides a broad review of the common and potentially serious DDIs between SACT medicines and other medicines taken in a cohort of lung cancer patients. It also confirms the lack of established procedure for Med-Rec in these patients and highlights the scope for improvement in identifying more potential interactions, particularly as cancer patients are at risk of polypharmacy. However, the multidisciplinary care required in oncology, including general practice, must be taken into account; thus, implementing these processes in a hospital setting would not eliminate all risk of drug-related harm, but rather, contribute to the bigger picture of ensuring safe medicine prescribing across the whole of the patient journey.

## Ethical considerations

Ethics approval is not required for this work as it is part of a service evaluation and improvement project. An ethics waiver was given by Chelsea and Westminster Hospital NHS Foundation Trust Research and Development lead and NRES for CLAHRC NWL Medicines Optimisation projects.

## Figures and Tables

**Table 1.** Summary of anticancer agents used in lung cancer with indications, class and mechanism of action. Taken and adapted with kind permission from [6], contributions from [1, 2, 4, 7–15].

Anticancer agent	Indication(s) in lung cancer	Class of anticancer agent	Main mechanism
Cyclophosphamide	Extensive SCLC	Nitrogen mustard	Intrastrand cross linking of DNA
Carboplatin, cisplatin	Stage I–II NSCLC (adjuvant)	Platinum-based compound	
	Stage IIIb–IV NSCLC (palliative)		
	All-stage SCLC (palliative)		
Pemetrexed	Stage IIIb–IV non-squamous NSCLC (first line)	Folate antagonist	Blocking the synthesis of DNA and/or RNA
Gemcitabine	Stage IIIb–IV squamous NSCLC (first-line)	Pyrimidine pathway antimetabolite	
Doxorubicin	Extensive SCLC	Anthracycline	Multiple effects on DNA/RNA synthesis and topoisomerase action
Docetaxel, paclitaxel	Stage IIIb–IV NSCLC (first line)	Taxane	Microtubule assembly; prevents spindle formation
Vincristine	Extensive SCLC	Vinca alkaloid	
Vinorelbine	Stage IIIb–IV squamous NSCLC (first line)		
Topotecan	Relapsed SCLC	Campothecin	Inhibition of topoisomerase
Etoposide	All-stage SCLC (palliative)	Other plant derivative	
Afatinib, erlotinib, gefitinib	Stage IV–NSCLC + EGFR mutation (first line)	Epidermal growth factor receptor (EGFR)-tyrosine kinase inhibitor (TKI)	Inhibition of kinases involved in growth factor receptor transduction
	Stage IV NSCLC (second line if refractory)		
Crizotinib	Stage IIIb–IV NSCLC + ALK translocation (second line)	Anaplastic lymphoma kinase (ALK) inhibitor	
Nintedanib	Stage IIIb–IV non-squamous NSCLC (second line, if progressive disease after first line)	Vascular endothelial growth factor receptor (VEGFR1-3), fibroblast growth factor receptor (FGFR1-3) and platelet-derived growth factor receptor (PDGFRα,β) inhibitor	
Nivolumab	Stage IV squamous and non-squamous NSCLC	Anti-programmed cell death-1 (PD-1) monoclonal antibody	Recruitment of T cells

**Table 2.** SACT regimens of all patients (n = 23).

SACT regimen	Frequency (%)
**Intravenous**	**14 (60.9)**
– Pemetrexed (maintenance)	– 7 (30.4)
– Post-pemetrexed + cisplatin	– 6 (26.1)
– Post-gemcitabine + carboplatin	– 1 (4.3)
– Nivolumab	– 3 (13.0)
– Gemcitabine + carboplatin	– 2 (8.7)
– Etoposide + carboplatin	– 1 (4.3)
– Pemetrexed + carboplatin	– 1 (4.3)
**Oral**	**7 (30.4)**
– Gefitinib	– 4 (17.4)
– Erlotinib	– 2 (8.7)
– Crizotinib	– 1 (4.3)
**Combined (intravenous and oral)**	**2 (8.7)**
– Carboplatin + vinorelbine	– 1 (4.3)
– Docetaxel + nintedanib	– 1 (4.3)

**Table 3.** Summary of the identified PDDIs (*n*=30). Key: UGT=UDP-glucuronosyltransferase; ACEIs=angiotensin-converting enzyme (ACE) inhibitors; CCBs=calcium channel blockers; ARBs=angiotensin-II receptor blockers; PPIs=proton pump inhibitors.

SACT medicine	Interacting (class of) drug	Possible outcome (effect of interaction)	Severity of interaction	n
Aprepitant	Ritonavir	Aprepitant toxicity (increased exposure)	Non-serious	1
	Warfarin	Reduced anticoagulation (reduced exposure to warfarin)	Non-serious	1
Crizotinib	UGT substrates (amoxicillin, colecalciferol, diazepam, levomepromazine, metoclo-pramide, mirtazapine)	Various (increased exposure to UGT substrates)	Unknown	6
	Dexamethasone	Poor crizotinib efficacy (reduced exposure)	Unknown	1
Dexamethasone	Antihypertensives (ACEIs, CCBs, ARBs, beta-blockers, nitrates)	Raised blood pressure (antagonised effect of antihypertensives)	Non-serious	11
	Antidiabetics (metformin, gliclazide, linagliptin)	Raised blood glucose (antagonised effect of antidiabetics)	Non-serious	6
	Diuretics (furosemide, bendroflumethiazide)	Hypokalaemia and associated signs and symptoms	Non-serious	5
	Aspirin	Gastrointestinal bleeding and ulceration (reduced exposure to salicylate)	Non-serious	5
	Calcium carbonate	Hypocalcaemia and associated signs and symptoms (reduced exposure to calcium salts)	Non-serious	4
	Coumarins	Enhanced (high-dose corticosteroids) or reduced anticoagulation (increased or reduced exposure to coumarins)	Potentially serious	1
	Phenindione	Enhanced or reduced anticoagulation (increased or reduced exposure to phenindione)	Non-serious	1
	Ritonavir	Adrenal suppression (increased exposure to corticosteroids)	Potentially serious	1
Docetaxel	CYP3A inhibitors (paracetamol, PPIs)	Docetaxel toxicity (increased exposure)	Unknown	2
	Clarithromycin	Myelosuppression; docetaxel toxicity (increased exposure)	Potentially serious	1
Domperidone	Opioid analgesics (codeine, morphine)	Gastroparesis (antagonised gastrointestinal effects of domperidone)	Non-serious	5
	Clarithromycin	Domperidone toxicity (increased exposure to domperidone); ventricular arrhythmias	Potentially serious	2
	Tiotropium	Gastroparesis (antagonised gastrointestinal effects of domperidone)	Non-serious	1
Erlotinib	PPIs/H2 antagonists/antacids (lansoprazole, ranitidine, sodium bicarbonate)	Poor efficacy (reduced exposure to erlotinib)	Unknown	3
	Statins	Myopathy	Unknown	1
Folic acid	Sodium bicarbonate	Folate deficiency and associated signs and symptoms (reduced exposure to folic acid)	Non-serious	2
Gefitinib
	CYP3A4 inhibitors (diclofenac, clindamycin)	Gefitinib toxicity (increased exposure)	Unknown	2
	PPIs (lansoprazole, omeprazole)	Poor gefitinib efficacy (reduced exposure)	Unknown	2
	CYP3A4 inducers (nevirapine, flucloxacillin)	Poor gefitinib efficacy (reduced exposure)	Unknown	2
	Warfarin	Enhanced anticoagulation (increased exposure to warfarin)	Potentially serious	1
Nivolumab	Dexamethasone	Systemic immunosuppression	Unknown	1
Ondansetron	Sertraline	Enhanced serotonergic effects	Non-serious	1
Pemetrexed	Nephrotoxic drugs (ibuprofen, ACEIs, sulphamethoxazole)	Nephrotoxicity; pemetrexed toxicity (increased exposure)	Non-serious	6
Platinum-based compounds (cisplatin, carboplatin)	Nephrotoxic drugs (aspirin, ibuprofen, ACEIs, sulphamethoxazole)	Nephrotoxicity	Unknown	7
	Diuretics (bendroflumethiazide, furosemide)	Nephrotoxicity; ototoxicity	Non-serious	5
Potassium chloride	Irbesartan	Hyperkalaemia and associated signs and symptoms	Potentially serious	1

**Table 4.** Summary of acknowledged and documented instances of PDDIs (*n* = 14).

Patient #	SACT medicine	Interacting medicine	Effect of interaction	Severity of interaction
1	Aprepitant	Ritonavir	Increased exposure to aprepitant	Non-serious
	Dexamethasone	Ritonavir	Increased exposure to corticosteroids, causing increased risk of adrenal suppression	Potentially serious
2	Nivolumab	Dexamethasone	Increased risk of systemic immunosuppression	Unknown
3	Aprepitant	Warfarin	Reduced anticoagulant effect of warfarin	Non-serious
	Dexamethasone	Coumarins	Enhanced (high-dose corticosteroids) or reduced anticoagulant effect of coumarins	Potentially serious
	Dexamethasone	Phenindione	Enhanced or reduced anticoagulant effect of phenindione	Non-serious
4	Gefitinib	Warfarin	Enhanced anticoagulant effect of warfarin	Potentially serious
5	Gefitinib	Lansoprazole	Reduced exposure to gefitinib	Unknown
6	Gefitinib	Flucloxacillin	Reduced exposure to gefitinib	Unknown
7	Erlotinib	Lansoprazole	Reduced exposure to erlotinib	Unknown
	Erlotinib	Statin	Increased risk of myopathy	Unknown
8	Gefitinib	Nevirapine	Reduced exposure to gefitinib	Unknown
	Gefitinib	Omeprazole	Reduced exposure to gefitinib	Unknown
	Gefitinib	Diclofenac	Increased exposure to gefitinib	Unknown

## Appendices

### Appendix 2. All anti cancer agents and supportive care medicines used as part of SACT regimens for treatment of the selected patient sample.

**Table:**

SACT regimen	Route of regimen	Supportive care medicines used	Route of supportive care medicine
Pemetrexed (maintenance)	Intravenous	Folic acid	Oral
		Ondansetron	Intravenous and oral
		Vitamin B12 (hydroxocobalamin)	Intramuscular
Pemetrexed + cisplatin	Intravenous	Aprepitant	Oral
		Dexamethasone	
		Domperidone	
		Folic acid	
		Magnesium sulphate	Intravenous
		Ondansetron	Intravenous and oral
		Vitamin B12 (hydroxocobalamin)	Intramuscular
Gemcitabine + carboplatin	Intravenous	Domperidone	Oral
		Dexamethasone	Intravenous and oral
		Ondansetron	
Nivolumab	Intravenous	None	N/A
Etoposide + carboplatin	Intravenous	Dexamethasone	Intravenous
		Ondansetron	
Pemetrexed + carboplatin	Intravenous	Aprepitant	Oral
		Dexamethasone	
		Domperidone	
		Folic acid	
		Magnesium sulphate	Intravenous
		Ondansetron	Intravenous and oral
		Vitamin B12 (hydroxocobalamin)	Intramuscular
Gefitinib	Oral	None	N/A
Erlotinib	Oral	None	N/A
Crizotinib	Oral	None	N/A
Carboplatin + vinorelbine	Intravenous and oral	Aprepitant	Oral
		Dexamethasone	Intravenous and oral
		Ondansetron	
		Magnesium sulphate	Intravenous
		Potassium chloride	
Docetaxel + nintedanib	Intravenous and oral	Domperidone	Oral
		Dexamethasone	Intravenous and oral
		Ondansetron	
